# Neurons run on fat

**DOI:** 10.1038/s44324-026-00130-4

**Published:** 2026-09-02

**Authors:** Rui Qi Teo, Gio Fidelito, Matthew J. Watt, Garron T. Dodd

**Affiliations:** Department of Anatomy and Physiology, The University of Melbourne, Melbourne, VIC Australia

**Keywords:** Biochemistry, Neuroscience

## Abstract

Neurons were long thought to depend primarily on glucose metabolism for ATP production. However, recent findings from three laboratories show neurons metabolise long-chain fatty acids as alternative fuel. Here, we highlight emerging evidence on the neutral lipid lipase DDHD2 and mechanisms enabling fatty acid mobilisation and oxidation in neurons. These advances reshape our understanding of neuronal bioenergetics and reveal an unexpected flexibility in how the brain can be fuelled.

## Introduction

Neurons primarily rely on glucose-derived substrates to generate ATP, with neuronal activity driving increased glucose uptake and glycolysis^1^. However, neurons have a limited capacity to store fuel (glucose)^2–4^, raising a key question of how energy demands are maintained when glucose availability is scarce. A widely described mechanism is the astrocyte-to-neuron lactate shuttle (ANLS), in which astrocytes supply metabolites, including lactate, to support neuronal metabolism^5–7^. However, this glucose-centric framework is now being revised in light of recent studies in *Nature Metabolism*^8–10^, demonstrating that neurons can also metabolise long-chain fatty acids as an alternative fuel, providing an additional route to maintain energy production under these conditions.

Fatty acid metabolism in the brain is mainly undertaken by glial cells. Notably, fatty acids in astrocytes are catabolised via mitochondrial β-oxidation and oxidative phosphorylation to support ATP production^11–13^. Fatty acids contribute substantially to brain energetics, accounting for an estimated 20% of total energy production, while also providing essential components of cellular membranes and signalling molecules^14^. Against this prevailing view, emerging evidence indicates that neurons are not so metabolically constrained, suggesting instead that they can take up long-chain fatty acids, sequester them in lipid droplets, and mobilise them for subsequent mitochondrial β-oxidation to sustain energy production when glucose is limited.

## Neurons store fatty acids in lipid droplets

Astrocytes canonically carry out the storage of lipid droplets in the brain in response to neuronal stress and to protect neurons against fatty acid-induced toxicity^11,15–17^. This process is carried out by fatty acid transporters and apolipoproteins (ApoE) to shuttle fatty acids from neurons to astrocytes, where they are stored as lipid droplets^11,15–17^. As astrocytes are highly glycolytic cells with major storage capacities for lipid droplets and glycogen, they are often described as the central hub of fatty acid metabolism and energy production in the brain^3,13^. This long-standing view supporting astrocytes’ roles in central lipid metabolism was first challenged in *Drosophila* with reports of significant lipid storage in neurons, and utilisation of lipid droplet-derived fatty acids for β-oxidation and energy production that was linked to memory function and learning^18,19^. The presence of lipid droplets in neurons has also been reported in mice^20^ demonstrating conservation of this response, however, the functional implications of neuronal lipid droplet storage and fatty acid metabolism remained unresolved.

**Fig. 1:**
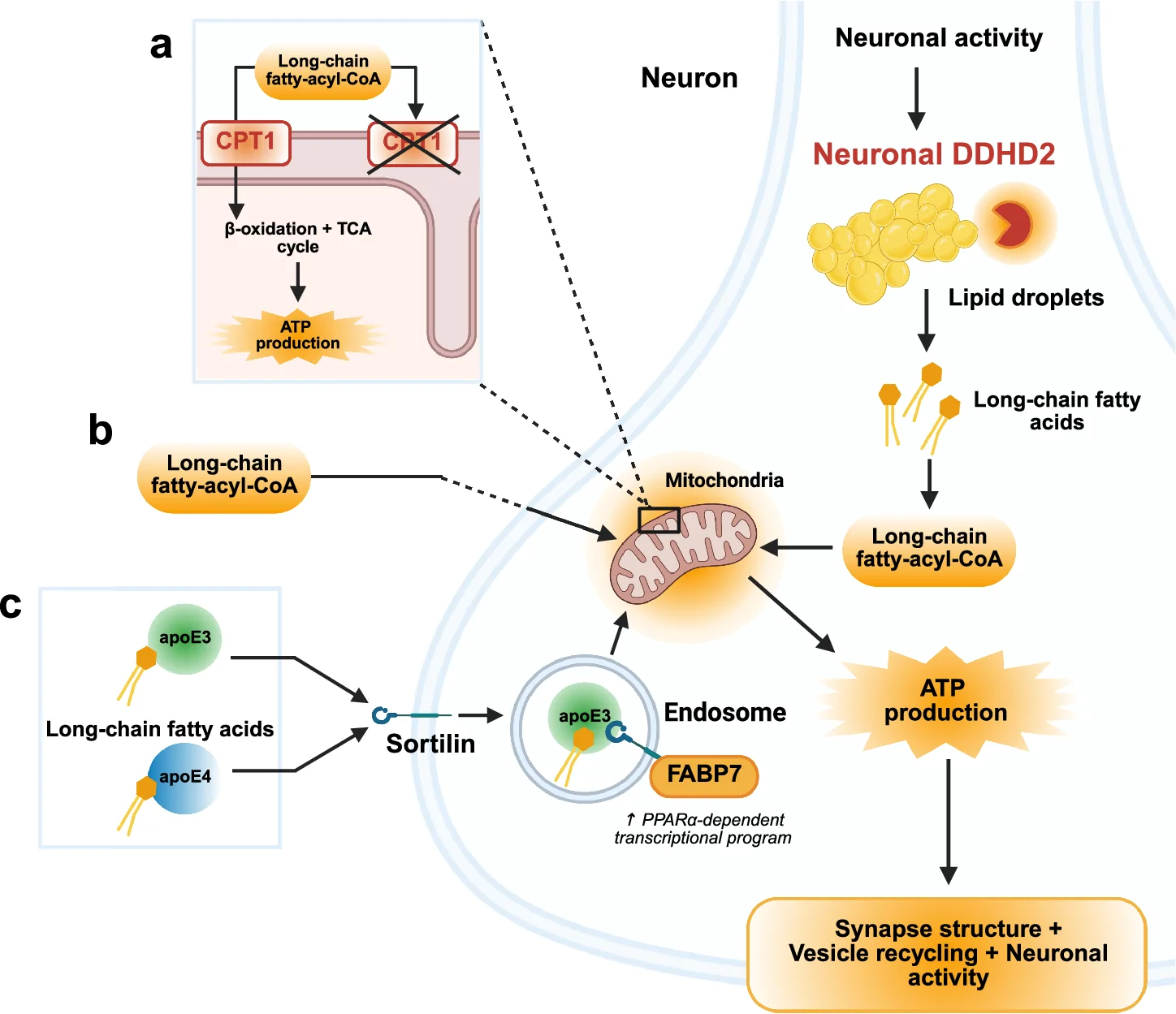
Neurons mobilise lipid stores to fuel mitochondrial β-oxidation and support synaptic function under glucose restriction. Neuronal lipid droplets are hydrolysed by the neutral lipid lipase DDHD2, liberating long-chain fatty acids that are activated to long-chain fatty-acyl–CoAs. **a** Fatty-acyl–CoAs are imported into mitochondria via CPT1 to drive β-oxidation and ATP production; this flux is reduced by pharmacological inhibition or genetic loss of DDHD2 or CPT1. **b** Exogenous supplementation with long-chain fatty-acyl–CoAs can rescue bioenergetic deficits caused by DDHD2 loss or inhibition. **c** apoE3, but not apoE4, interacts with sortilin to promote endosomal lipid uptake/trafficking; with assistance from FABP7, this pathway enhances delivery of long-chain (particularly polyunsaturated) fatty acids and engages a PPARα-dependent transcriptional program that increases mitochondrial fatty acid oxidative capacity. Collectively, these pathways highlight an underappreciated neuronal reliance on fatty acids as an alternative fuel when glucose is limited, and link lipid metabolism to vesicle recycling, synaptic integrity, and neuronal activity. Figure derived from findings by^8–10^. apoE3 apolipoprotein E3, apoE4 apolipoprotein E4, CPT1 carnitine palmitoyltransferase 1, CoA coenzyme A, FABP7 fatty acid-binding protein 7, TCA tricarboxylic acid. Created in BioRender. Teo, R. Q. (2026) https://BioRender.com/vl771rb

New research by Kumar et al.^8^ and Saber et al.^9^ addressed this knowledge gap in mammalian cells and mice. These studies explored the roles of DDHD Domain Containing 2 (DDHD2), a neuron-enriched neutral lipid lipase that mediates the hydrolysis of triglycerides and phospholipids, releasing fatty acids for cellular use^21,22^. Pathogenic variants of the DDHD2 gene cause hereditary spastic paraplegia 54 (HSP54), a disorder associated with neuromuscular and cognitive impairment^21–23^. Despite this genetic link, the mechanistic role of DDHD2 in mammalian neurons and its functional outcomes remained unclear. Kumar and colleagues first mapped DDHD2 expression to neurons in the hippocampus of the mouse brain, a region critical to learning and memory^8^. In hippocampal neuron cultures, DDHD2 is localised predominantly to presynaptic nerve terminals. Notably, pharmacological inhibition of DDHD2 led to neuronal lipid droplet accumulation, indicating a regulatory role for DDHD2 in triglyceride/fatty acid cycling in neurons (Fig. 1). This finding was consistent with preceding evidence in other cell lines (HeLa) harbouring the loss of DDHD2^22^. Together, these findings establish that lipid droplets are dynamically regulated in neurons. The presence of lipid droplets in neurons adds to our understanding that both astrocytes and neurons regulate lipid droplets, pointing to a more complex regulation of brain fatty acid metabolism than the prevailing dogma suggests.

## Fatty acids are mobilised and used by neurons for ATP production

The accumulation of lipid droplets at nerve terminals following DDHD2 inhibition prompted the question of whether neurons use fatty acids for energy production. Long-chain fatty acids are transported into the mitochondria by carnitine palmitoyl transferase 1 (CPT1), where they undergo β-oxidation to generate ATP. Consistent with this, CPT1 inhibition reduced neuronal ATP production (Kumar et al.^8^, Saber et al.^9^, and Greda et al.^10^), indicating that lipid droplet-derived or extracellular fatty acids can fuel mitochondrial β-oxidation when glucose is absent, a finding also observed in *Drosophila*^19^.

DDHD2 was previously shown to preferentially liberate the saturated fatty acids myristate (C14:0), palmitate (C16:0), and stearate (C18:0) from triglycerides and phospholipids^24^. Kumar et al.^8^ demonstrated using a pulse-chase assay that lipid droplet-derived fatty acids are transported into mitochondria, and separately, using a genetically encoded mitochondrial ATP sensor, that DDHD2 activity maintains mitochondrial ATP levels in the absence of glucose. Complementing these findings, Saber et al.^9^ showed that deletion of Ddhd2 reduced neuronal ATP production, which was restored by supplementation of myristoyl-CoA, palmitoyl-CoA, and stearoyl-CoA in the culture medium in a CPT1-dependent manner (Fig. 1b).

In contrast to the saturated fatty acids liberated intracellularly by DDHD2, the mechanism/s mediating the transport of extracellular fatty acids into neurons to maintain neuronal ATP production^9^ was previously unknown. Greda et al.^10^ explored one such mechanism, demonstrating that apolipoprotein E3 (apoE3), secreted by astrocytes and microglia, transports extracellular polyunsaturated fatty acids (PUFAs), a distinct lipid class from the saturated species above, to neurons, where they are internalised by the transmembrane protein sortilin (Fig. 1c). This in turn activates peroxisome proliferator-activated receptor (PPARα) expression and transcriptional activity, promoting mitochondrial ATP production. PPARα is a transcription factor and master regulator of lipid metabolism, well-characterised in the liver and heart^25^, and its involvement in neuronal lipid metabolism expands our understanding of PPARα’s importance to other cell types.

Overall, studies by Kumar et al., Saber et al., and Greda et al. provide complementary insights into how intracellular and extracellular fatty acids are mobilised and utilised by neurons through DDHD2-mediated mechanisms or the sortilin-ApoE3 pathway. Kumar et al. established that fatty acids derived from intracellular lipid droplets contribute to 30–50% of basal energy production in neurons at normal glucose levels, while Saber et al. demonstrated that DDHD2 specifically supplies saturated long-chain fatty acids (myristate, palmitate, stearate) required for synaptic vesicle trafficking and long-term potentiation, with functional rescue achieved by saturated, but not unsaturated, fatty acids. Greda et al. point to a complementary extracellular route by which neurons acquire fatty acids through astrocyte-to-neuron lipid transfer, with an apparent bias toward PUFA handling, activating PPARα-driven reprogramming to enhance fatty acid oxidation capacity. However, it is important to contextualise these in vitro findings within the broader landscape of neuronal energy metabolism during glucose scarcity. Ketone bodies, namely β-hydroxybutyrate and acetoacetate, have long been established as the dominant alternative fuel source for the brain during fasting and starvation^12,26,27^. Neurons have been shown to oxidise ketone bodies^28^, and in vivo evidence has demonstrated that ketone bodies fuel neurons during fasting to sustain associative memory formation^29^. The relative contribution of long-chain fatty acid oxidation to neuronal energy production during glucose scarcity therefore remains to be fully established in vivo, and future studies should examine the interplay between fatty acid and ketone body metabolism under physiological fasting conditions to better delineate their respective contributions to neuronal energy homeostasis.

## Linking metabolism to neural function

The convergent theme in all three studies is that neuronal fatty acid metabolism supports synaptic function, particularly vesicle recycling, which is essential for maintaining synaptic architecture and neuronal communication (Fig. 1). Using a pH-sensitive fluorescent reporter of exocytosis, vesicle recycling at synapses was reduced by pharmacological inhibition^8^ or genetic loss of DDHD2^9^. Kumar et al. further demonstrated that this metabolic support is activity-dependent: electrophysiological stimulation increased fatty acid flux from lipid droplets to mitochondria, whereas Na⁺ channel blockade reduced this transfer, indicating that DDHD2-dependent fatty acid mobilisation is coupled to synaptic demand (Fig. 1a).

Complementing these findings, Saber et al. demonstrated that DDHD2 supplies saturated long-chain fatty acids, specifically myristate and palmitate, that are essential for synaptic vesicle trafficking and long-term potentiation (LTP). Notably, functional rescue was achieved specifically by saturated, but not unsaturated fatty acids, highlighting the importance of fatty acid composition, rather than just availability, in supporting synaptic plasticity. This specificity may be explained, at least in part, by protein fatty acylation, whereby the covalent attachment of myristic and palmitic acids to proteins via N-myristoylation and S-palmitoylation, respectively, regulates protein trafficking, stability, and interactions^21^. Whether these phenotypes are directly attributable to protein fatty acylation, however, remains to be established.

In contrast, Greda et al.^10^ highlight an adaptive mechanism, where fatty acids act not only as fuels but also as signals that induce a transcriptional program to increase neuronal capacity for long-chain fatty acid oxidation. Disruption of the sortilin-apoE3 pathway in human neuron-glia cultures impaired neuronal activity when glucose was withdrawn, consistent with a failure to engage this polyunsaturated fatty acid-driven metabolic reprogramming. Collectively, these studies indicate that neuronal fatty acids contribute to brain function through two complementary modes: 1) as an acute support of synaptic energetics and membrane/trafficking demands, and 2) via transcriptional adaptation that expands oxidative capacity during nutrient limitation (Fig. 1).

## Unanswered questions and future directions

These studies provide new insight into how neurons utilise fatty acids to support energy production, synaptic structure, and neuronal function. As much of the mechanistic work was performed in vitro, there is a clear need to explore these findings in vivo, where substrate availability, circuit activity, and cell-cell lipid trafficking are dynamically regulated. Kumar et al. began to bridge this gap by inhibiting DDHD2 or CPT1 in mice, which increased susceptibility to torpor during nutrient scarcity. This supports an essential role for neuronal fatty acid metabolism under significant whole-body metabolic stress. The mouse models generated by Greda and colleagues could serve as models of neuronal metabolic inflexibility, that is, an inability to switch fuel use appropriately between carbohydrates and fats in response to changes in nutritional state, hormonal signals, or energy demands. In a recent study, the presence and functional significance of neuronal lipid droplets were expanded in invertebrates (*Drosophila* and *Caenorhabditis elegans*) and mice^30^. Given the sex, regional, and functional heterogeneity across species in current studies^8,9,19^, future studies assessing how these models respond to metabolic and behavioural challenges and effects on cognition are warranted to pinpoint the necessity of neuronal fatty acid metabolism.

The canonical view that fatty acids are shuttled from neurons to astrocytes is largely driven by the need to protect neurons from ROS generation, lipid peroxidation, and excessive ceramide production^11,30^. If neurons actively oxidise fatty acids, the mechanisms protecting them from lipotoxicity warrant careful consideration. The tight regulation of DDHD2-mediated lipolysis may couple fatty acid release to immediate CPT1-mediated translocation, limiting cytoplasmic accumulation. Similarly, the sortilin-apoE3-PPARα axis may serve a dual purpose: not only facilitating fatty acid uptake and utilisation for energy production, but also safeguarding against lipotoxicity by transcriptionally upregulating genes encoding key regulators of fatty acid oxidation, ensuring that free fatty acids are efficiently channelled into mitochondrial β-oxidation. Nevertheless, how neurons manage fatty acid toxicity during active oxidation remains an important open question for future studies.

DDHD2 has been investigated in the hippocampus due to its abundant expression and relevance in learning, memory, and behaviour^19,24^. According to the Human Protein Atlas (proteinatlas.org)^31^, DDHD2 expression extends beyond the hippocampus, raising important questions about whether DDHD2-dependent lipolysis plays region- and cell type-specific roles across the brain. More broadly, multiple genes encoding key regulatory enzymes of fatty acid metabolism show heterogeneous expression across brain regions^32,33^, suggesting that the capacity for fatty acid uptake, storage, and oxidation may vary across neuronal populations and brain regions. This heterogeneity creates a compelling framework for exploring neuronal lipid dysregulation in neurological disorders, where altered substrate use or excessive reliance on fatty acids could contribute to hyperexcitability and neurotoxicity^34–36^. In this context, it will be informative to test whether DDHD2 overexpression enhances neuronal lipid turnover and protects against neuropathology driven by chronic lipid accumulation, and whether any protective effects are restricted to specific circuits or neuronal subtypes.

Other routes of fatty acid delivery to neurons should also be considered. For example, neurons can facilitate long-chain fatty acid uptake via the fatty acid translocase CD36^37^. In the study by Greda et al., binding of fatty acid–binding protein 7 (FABP7) to the sortilin-apoE3 endosomal complex is required for transport of PUFAs and subsequent PPARα-dependent transcriptional reprogramming (Fig. 1c). Because FABP7 and CD36 also participate in astrocyte-to-neuron lipid transfer^37,38^, future work should broaden beyond a single pathway to map the breadth of fatty acid uptake and trafficking mechanisms across cell types and physiological states.

Although Greda et al. present evidence that this pathway is neuron-intrinsic, it raises the possibility that glia may indirectly gate DDHD2-dependent lipid use by controlling apoE availability, lipid delivery, and metabolic cues. Reactive or inflammatory glial states could modify apoE3 expression, secretion, and lipidation, thereby reshaping neuronal fatty acid uptake and utilisation. Moreover, evidence for bidirectional lipid trafficking between neurons and astrocytes^11,39^ supports a dynamic, multi-directional crosstalk that maintains brain lipid homeostasis, rather than a simple one-way shuttle.

Active triglyceride–fatty acid cycling in neurons raises a key question: how do neurons meet their fatty acid requirements in vivo, and does the availability and composition of free fatty acids shift across physiological states and in metabolic diseases, such as obesity or starvation^40^. In line with Kumar et al., knocking out DDHD2 led to lipid droplet accumulation, suggesting that neurons can either import fatty acids and/or generate them via de novo lipogenesis, but fail to efficiently mobilise stored lipid. Importantly, Kumar et al. also showed that extracellular fatty acids can be taken up by neurons to sustain mitochondrial respiration and ATP production, supporting the concept that circulating or locally supplied fatty acids may be an important substrate for DDHD2-driven lipolysis, particularly when carbohydrate-derived fuels are constrained.

## Conclusions

Collectively, these studies uncover previously underappreciated roles for neuronal fatty acid uptake, storage, and oxidation, refining our view of how neurons sustain bioenergetic output when canonical substrates such as glucose and lactate are constrained. Importantly, they position neuronal lipid metabolism as a driver not only of ATP production, but also of fundamental aspects of synaptic maintenance and neuronal function. A key next step is to ascertain how much neuronal energy demand is met by fatty acid oxidation, and to define when and where this contribution becomes physiologically meaningful in vivo (e.g., across brain regions, activity states, fasting/feeding, and ageing). Establishing the magnitude and context-dependence of this pathway could reshape how we think about neuronal bioenergetics and open new therapeutic avenues for disorders in which metabolic flexibility fails.

## Data Availability

No datasets were generated or analysed during the current study.
